# Giant fibrous hamartoma of infancy: A case report

**DOI:** 10.1097/MD.0000000000019489

**Published:** 2020-03-13

**Authors:** Sun Wang, Qichao Ma, Hao Ying, Qin Jiao, Dan Yang, Bin Zhang, Lihua Zhao

**Affiliations:** a Department of Orthopedics, Shanghai Children's Hospital, Shanghai Jiao Tong University, Shanghai, China; b Department of Pathology, Shanghai Children's Hospital, Shanghai Jiao Tong University, Shanghai, China.

**Keywords:** differential diagnosis, fibrous hamartoma of infancy, prognosis, treatment

## Abstract

**Rationale:**

Fibrous hamartoma of infancy (FHI) is a rare benign soft-tissue tumor in children with a triphasic organoid histologic appearance.

**Patient concerns:**

We here report a case with the largest FHI published so far. A 13-month-old boy with a rapidly growing tumor 45 cm in length and 69.3 cm in circumference of the left lower extremity was identified immediately at birth.

**Diagnoses:**

The diagnosis was uncertain before the operation, although biopsy was conducted. The postoperation histological examination showed arranged spindle cells, adipose tissue, and nests of immature small cells embedded in a myxoid background, which is the characteristic triphasic histology of FHI.

**Interventions:**

Under general anesthesia, hip and lower extremity amputation was performed.

**Outcomes:**

The patient was followed-up for 6 years and no signs of recurrence were found, suggesting a favorable prognosis, although a part of the residual tumor was present after the surgery.

**Lessons:**

FHI exhibits pathological and clinical characteristics. However, differential diagnosis of FHI from other soft-tissue tumors before operation remains a challenge. Thus, sometimes, aggressive therapy for the treatment of FHI might be a good choice to get a satisfactory.

## 1 Introduction

Fibrous hamartoma of infancy (FHI) was initially defined as “subdermal fibrous tumor of infancy” by Reye in 1956.^[1]^ In 1965, FHI became the currently accepted nomenclature.^[2]^ FHI is a benign mesenchymal lesion of myofibroblastic origin with prototypical triphasic histopathological features and mesenchymal, fibrous composition. Characteristic fatty tissue elements can be observed in variable proportions, in the absence of anaplasia and/or mitoses.^[3]^

FHI typically occurs in the first 2 years of life, predominantly in males, and the male/female ratio is 2.0 to 2.4:1.^[4,5]^ The disease may occur in various tissues of the body; it is most frequently localized in the upper extremities, axilla, shoulders, groin, upper trunk and sacral regions.^[5–11]^ Sometimes, it is so difficult to differentiate FHI from other soft tissue tumors before surgery that we cannot obtain appropriate treatment option.

We herein present a case of FHI that the lesion size of this being one of the largest reported so far. The 6 years’ follow-up shows that FHI may not recur even though the resection of the tumor is not complete.

## 2 Patient information

**Figure 1:**
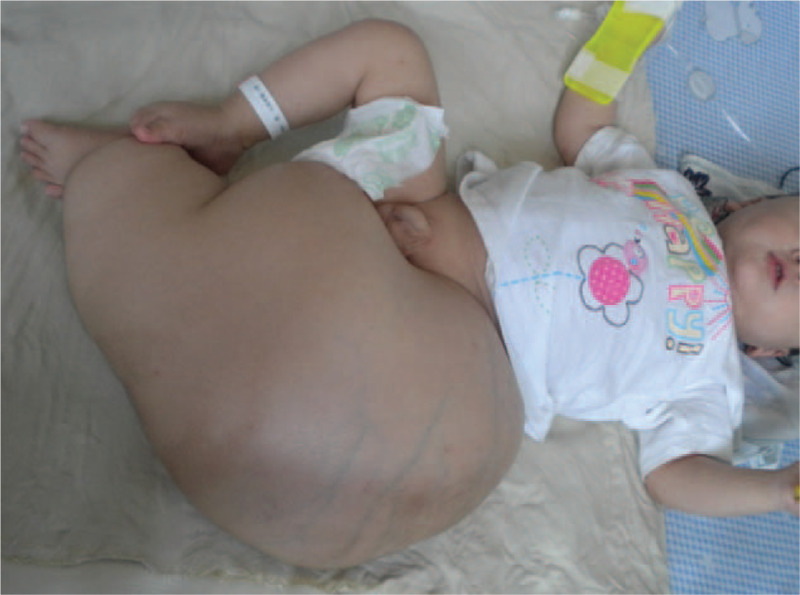
A 13-month-old boy with fibrous hamartoma of infancy, which is 45.0 cm in length and 69.3 cm in circumference in the left lower extremity (anterior view).

**Figure 2:**
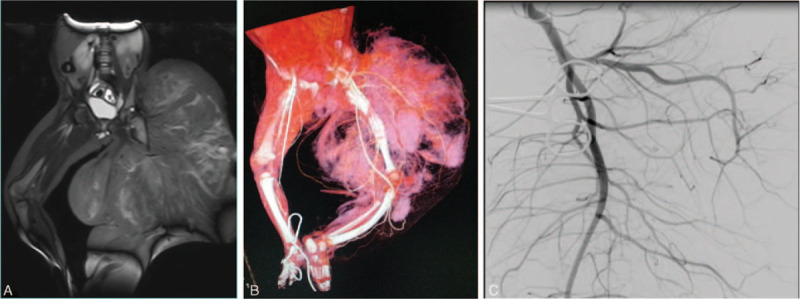
The preoperative MRI, CTA, and DSA examination of the patient. (A) The Axial and coronal MRI image demonstrated a giant mass with ill-defined border. The musculature, fascia, overlying subcuticular fat and skin infiltration was obvious. (B and C) CTA and DSA demonstrated increased and abnormal morphology of vascular branches.

**Figure 3:**
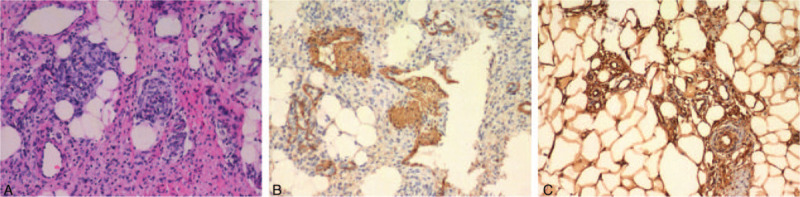
Histological findings. (A) Hematoxylin-eosin (H&E) staining indicated the classic histopathologic features of FHI (×200). (B and C) Immunohistochemical specimens for SMA and CD34, respectively (×200).

A 13-month-old boy presented to our orthopedic department due to prominent increased diameter and length of the left lower extremity that presented at birth. Physical examination showed a painless, hard, giant left lower extremity, 45.0 cm in length and 69.3 cm in circumference, which was much bigger and longer than the right lower extremity (Fig. 1). The overlying skin was normal. The left leg was immobilized due to the giant rapidly growing mass. Signs of the chronic, neoplastic condition or endocrine abnormality were not detected. All signs including birth, gestational, maternal, and family histories of this patient were normal. In addition, the patient's developmental history and growth curves and laboratory examinations were normal. Intravenous contrast-enhanced magnetic resonance imaging (MRI) showed a heterogeneous, no capsule, infiltrative tissue formation invaded into the muscular structures and vascular-nervous bundles, with ill-defined and therefore difficult to measure borders (Fig. 2A). Computed tomography angiography (CTA) (Fig. 2B) and digital subtraction angiography (DSA) (Fig. 2C) showed a much longer and bowing femur and tibial of the left lower extremity that had plentiful collateral circulation. Because of the giant and constant rapid growing tumor, under demand of patient parents, amputation of the left hip and extremity surgical intervention was performed to eliminate the tumor as much as possible. Intraoperatively, surgeons attempted to include a 0.5 cm margin of normal tissue in order to prevent tumor recurrence. However, the resection was limited because of the infiltration of the tumor into vital structures. A large lesion was excised with multiple tissue fragments of white and/or yellow appearance and no normal muscles in the tumor. Dimensions of excised lesion were 38 × 29 × 18 cm with ill-defined border and no necrosis. The biopsy specimen demonstrated myxoid cellular areas admixed with fibroadipose tissue. Microscopically, the tissue fragments were composed of randomly arranged spindle cells with elongated bland-looking nuclei admixed with vascular channels. Mature adipose tissue interspersed with bands of dense fibrous tissue rich in myofibroblasts and collagen, and nests of immature mesenchyma in a myxoid background. The mesenchymal cells were small or medium-sized and spindle or round-shaped in morphology (Fig. 3A). The overall cellularity was moderate and no evidence of mitosis, necrosis, and/or atypia. Immunohistochemical analysis demonstrated positivity for smooth muscle actin (SMA) and CD34 (Fig. 3B, C). On the basis of those findings, the definitive diagnosis of FHI was made.

No postoperative complications occurred. At the 6-year postoperative follow-up, the patient recovered well and has a satisfactory life using an artificial left lower extremity, and no signs of tumor recurrence according to the results of physical examination and MRI.

## 3 Discussion

The children affected by FHI typically present with no specific symptoms.^[3,4]^ As shown in the literature, most of the FHI are slow-growing, but it may also present as a rapid growing mass with signs of warmth, tenderness, and pain.^[3–5]^ Our case presented a painless, hard, no warmth, and rapid growing mass. Overlying hyperpigmentation, hyperhidrosis, and/or hypertrichosis have also been reported, but these signs are not presented in our case.^[12]^ Most of the lesions are small and solitary, the size ranging from 0.5 to 9.0 cm.^[3–12]^ The largest tumor diameter reported in the literature is 20 cm in a 7-year-old Nigerian girl. However, her diagnosis of FHI was questioned.^[13]^ In our case, the tumor size was larger than 29 cm in diameter, which is the largest FHI reported to date.

The main challenge for us is to differentiate FHI from other soft-tissue tumors such as neurofibrimas, vasvular tumors, juvenile fibromatosis, and sarcoma, especially when the tumor is hard and fixed to the deep planes. A careful histological examination by routine hematoxylin-eosin staining can provide sufficient indications for a correct diagnosis. The histological criteria for diagnosis of FHI included the presence of well-defined bundles of dense, intersecting fascicles of fibroblasts/myofibroblasts in the collagenous stroma, and the nests of immature oval or stellate cells in the basophilic mucoid stroma, admixed with mature adipose tissue. The immature component does not harbor significant atypia, pleomorphism, and/or aggressiveness.^[3]^ A sign of fibrous tissue trabeculae interspersed with fat in an organized pattern helps narrow down the differential diagnosis, which is strongly suggestive of FHI. Immunohistochemistry can assist the identification of the tumor origin. The histological staining of our case demonstrated mature adipose tissue intersected bands of dense fibrous tissue rich in myofibroblasts and collagen, and nests of immature mesenchyma in a myxoid background. Although differential diagnosis of FHI is difficult, the histological manifestations of our case meet the histological diagnosis criteria for FHI described above. Positive immunohistochemical staining for SMA and CD34 proteins was noted in our case, which support the diagnosis of FHI.

The preferred treatment for FHI is complete local resection. Park et al^[14]^ reported that tyrosine kinase inhibitors may be used as adjunctive therapy in the case of resection is particularly difficult. Repeated and nondestructive surgery is suggested to medical practitioners. Local and conservative excision is usually conducted notably in symptomatic patients and the surgical procedures are often curative.^[4,5]^ Majority of the studies indicated that aggressive, mutilating resections should be avoided due to the excellent overall prognosis and low recurrence rate, although some studies recommended local radical excision in order to prevent accelerated spread of the tumor.^[15]^ In the present report, a treatment strategy of destructive amputation of the left hip and the left lower extremity was forced to be selected because of the giant size, infiltration, rapid growth of the tumor, and the immobility of the left leg. Now, the patient can walk and play like a normal child with the help of artificial extremity. The tumor did not recur after 6 years of follow-up. It has been reported that the postoperative recurrence incidence is 16.7% in cases of giant fibrous hamartomas at 42 months following primary excision.^[4]^ Typically, a gap between the normal tissue and the periphery of tumor should be taken to prevent the spread of the tumor cells.^[3,4]^ McGowan et al^[15]^ reported 2 cases of FHI. Positive initial resection margins in one case correlated with rapid postoperative recurrence, whereas complete excision with a 0.5-cm margin in the second case did not result in recurrence of the tumor. However, Agrawal et al^[16]^ found that the margin status may not influence the propensity of clinical recurrence. The result of our case is consistent with that of Agrawal et al.^[16]^ So far, no recurrence has been found, although the tumor has not been completely resected. The follow-up period in our case is short and a longer follow-up is required, as previous studies have reported an FHI recurrence 14 years after the primary excision.^[17]^ No metastases, histological regression, involution, and/or malignancy have been reported for FHI to date.

FHI exhibits its special pathological and clinical characteristics. The differential diagnosis of FHI from other soft tissue tumors before operation remains a challenge, which is what we should pay attention to. Sometimes, we have to choose aggressive therapeutic approaches for the treatment of FHI when the tumor is very big and deep to get a satisfactory life quality.

## Acknowledgments

We are grateful for unrestricted support and enthusiasm of the families involved in this report. This manuscript is dedicated to them, who with all adversity never lost their hope.

## Author contributions

**Conceptualization:** Lihua Zhao.

**Data curation:** Sun Wang, Qichao Ma, Qin Jiao, Bin Zhang.

**Formal analysis:** Hao Ying.

**Investigation:** Lihua Zhao, Sun Wang.

**Methodology:** Lihua Zhao, Sun Wang.

**Resources:** Hao Ying, Lihua Zhao.

**Validation:** Hao Ying, Lihua Zhao.

**Visualization:** Bin Zhang.

**Writing – original draft:** Qichao Ma, Lihua Zhao, Qin Jiao.

**Writing – review & editing:** Lihua Zhao, Qichao Ma.
